# Understanding Characteristics, Treatment Patterns, and Clinical Outcomes for Individuals with Advanced or Recurrent Endometrial Cancer in Alberta, Canada: A Retrospective, Population-Based Cohort Study

**DOI:** 10.3390/curroncol30020176

**Published:** 2023-02-14

**Authors:** Diana Martins, Dylan E. O’Sullivan, Devon J. Boyne, Winson Y. Cheung, Odette Allonby, Mara Habash, Darren R. Brenner, Justin Riemer, Jacob McGee

**Affiliations:** 1 GSK, Mississauga, ON L5R 4H1, Canada; 2 Oncology Outcomes Initiative, Department of Oncology, Cumming School of Medicine, University of Calgary, Calgary, AB T2N 1N4, Canada; 3 Department of Obstetrics and Gynecology, Schulich Medicine and Dentistry, Western University, London, ON N6A 5C1, Canada

**Keywords:** advanced/recurrent endometrial cancer, treatment patterns, Canada, platinum-based chemotherapy, treatment outcomes

## Abstract

Endometrial cancer (EC) incidence has increased in recent decades. However, population-based outcomes data are limited. In this retrospective cohort study, we examined characteristics, treatment patterns, and clinical outcomes, including time to next treatment (TNNT) and overall survival (OS), among advanced/recurrent (A/R) EC patients between 2010 and 2018 in Alberta, Canada. Kaplan–Meier statistics evaluated TTNT and OS, stratified by patient (A/R) and treatment. A total of 1053 patients were included: 620 (58.9%) advanced and 433 (41.1%) recurrent. A total of 713 (67.7%) patients received first-line therapy: 466 (75.2%) advanced and 247 (57.0%) recurrent. Platinum-based chemotherapy (PBCT) was the most common first-line regimen (overall: 78.6%; advanced: 96.1%; recurrent: 45.3%). The median TTNT and OS from first-line therapy were 19.9 months (95% confidence interval [CI]: 17.5–23.5) and 35.9 months (95% CI: 31.5–53.5), respectively. Following first-line PBCT, the median OS from second-line chemotherapy (N = 187) was 10.4 months (95% CI: 8.9–13.3) and higher for those rechallenged with PBCT (N = 72; 38.5%) versus no rechallenge (N = 115; 61.5%) (13.3 months [95% CI: 11.2–20.9] vs. 6.4 months [95% CI: 4.6–10.4; *p* < 0.001]). The findings highlight poor outcomes in A/R EC, particularly following first-line therapy, and that additional tolerable therapeutic options are needed to improve patient outcomes.

## 1. Introduction

Endometrial cancer (EC) is the fourth most common cancer among Canadian women, and the most common gynecologic malignancy, with an earlier estimate of nearly 8100 new cases projected to be diagnosed in 2022 [1]. Concerningly, the incidence of EC in Canada has been increasing in recent years, similar to that observed globally [2,3]. EC mortality rates in Canada are also increasing, with an earlier estimate of approximately 1500 deaths due to EC in 2022 alone [1]. Survival outcomes for women who present with advanced EC or who experience a recurrence of their early stage disease are poor, and the estimated five-year survival is approximately 18.4% for those with metastatic disease [4]. The poor outcomes in patients with advanced/recurrent EC are largely due to the limited efficacy of chemotherapy in this setting and the lack of meaningful improvements in the treatment paradigm over the past decades [5].

Current treatment modalities for EC include chemotherapy and radiotherapy, and for patients with low-grade endometrioid hormone-receptor (HR)-positive (i.e., type-I) histology, hormone therapy is an option [6,7,8]. European guidelines on currently accepted approaches for the treatment of patients with EC have been published in the past few years (2021) [8]; however, there are few consensus-based guidelines for EC that exist in Canada [6,9]. Patients with advanced/recurrent disease are generally recommended for treatment with platinum-based doublet chemotherapy in the first line [6,8,10], although this regimen is associated with a high degree of toxicity, including but not limited to gastrointestinal issues, alopecia, white blood cell toxicity, thrombocytopenia, anemia, and peripheral neuropathy [11,12]. While there is no established standard of care for the treatment of patients with advanced/recurrent EC following progression on platinum-based chemotherapy (PBCT), including both platinum monotherapy and combination therapies (e.g., cisplatin or carboplatin alone or in combination with other agents), rechallenge with PBCT has been associated with favorable outcomes in certain patients [13]. Sensitivity to retreatment with PBCT (based on a platinum-free interval) has been investigated in several studies and may guide treatment decisions in these patients [14,15]. Given the limited efficacy of available chemotherapies, clinical trials are also considered for patients who are eligible [6,9]. These ongoing trials are focused on personalized treatment strategies based on histopathologic and molecular factors of EC [16,17]. Outside of the clinical trial setting, patients generally receive palliative treatment with single-agent chemotherapies, hormone therapy, or end-of-life care [7,9,18].

Recently, targeted immunotherapies such as programmed cell death protein 1 (PD-1) inhibitors have emerged, with the potential to change the treatment landscape for patients with advanced and recurrent EC. These PD-1 inhibitors block binding to programmed-death ligands (PD-L1 and PD-L2), resulting in the release of inhibition of PD-1-pathway-mediated immune responses, including the antitumor immune response [19]. PD-1 inhibitors, pembrolizumab and dostarlimab, have recently been approved as monotherapy by Health Canada (HC) for the treatment of adult patients with mismatch repair deficient (dMMR) EC [20,21,22,23]. In addition, lenvatinib in combination with pembrolizumab has been approved by HC for the treatment of adult patients with advanced EC that is not microsatellite instability–high or dMMR [24,25].

Although medications approved by HC are permitted to be marketed in Canada, access to these treatments is currently limited because the cancer drug regulatory and public drug funding process is lengthy and complex [26,27]. Given that the treatment landscape in Canada for advanced and recurrent EC patients is evolving, it is important to describe current real-world treatment patterns and clinical outcomes to highlight the value that novel therapies and emerging evidence may have for future patients.

In this study, we examined patient characteristics, treatment patterns, and clinical outcomes, such as time to next treatment (TTNT) and overall survival (OS) by the line of therapy, among women with advanced/recurrent EC in Alberta, Canada.

## 2. Materials and Methods

### 2.1. Setting and Design

We conducted an observational, population-based, retrospective cohort study among adult females (≥18 years of age) in Alberta, Canada, newly diagnosed with advanced EC (de novo stage IIIB, IIIC, or IV) or recurrent EC (recurrence from de novo stage I, II, or IIIA) between January 2010 and December 2018, with follow-up to December 2019.

### 2.2. Data Sources

This investigation relied upon various provincial administrative databases that provide coverage for the entire population of Alberta, Canada. Included are a total of 17 cancer centers (2 tertiary, 4 regional, and 11 community hospitals) from a publicly funded health system. Every resident of Alberta is assigned a unique lifetime identifier upon becoming a resident of the province, which is used to link demographic information, healthcare encounters, and electronic medical records in the province from various databases. The Alberta Cancer Registry was used to identify all diagnoses of EC, the histology, stage of cancer and age at diagnosis, date of death, and cause of death. The ARIA electronic medical record database was used to capture information on treatments (surgery, chemotherapy, hormone therapy, radiation, and treatment location). Hospitalizations, emergency room visits, and physician office visits were ascertained from the Discharge Abstract Database, National Ambulatory Care Reporting System Database, and Physician Claims Database, respectively. Due to the ability to capture data from all treatment settings in Alberta, all patients who were identified as being diagnosed with primary advanced endometrial cancer were included in this dataset, regardless of referral status.

### 2.3. Cohort Creation

We included all females 18 years of age or older with a diagnosis of type I (low or unknown grade and endometrioid carcinoma) and type II (high grade or serous/clear/mixed histology) EC. Advanced EC was defined as individuals with a confirmed diagnosis of advanced-stage IIIB/IIIC/IV EC (Figure S1). As recurrent disease is not captured in the databases available, recurrent EC was defined by an algorithm based on the work of Xu et al. (2019) [28] as patients with an early stage diagnosis (I, II, or IIIA) of EC and who had 2+ cycles of chemotherapy, radiation, or death due to EC that occurred more than a year after their first treatment (surgery, radiation, or systemic therapy) date. Additionally, patients with an initial diagnosis of early stage type I EC were defined as having recurrent disease if they received hormone therapy more than two years after the date of their first treatment, which would be indicative of a hormone-related treatment for disease progression and in line with common treatment practice for Type I (but not type II) recurrent EC in Canada [6,29] A two-year window was used to avoid capturing hormone use as maintenance therapy. Patients with missing tumor type characteristics were classified as type II if they had evidence of grade 3 disease or type I if they had grade 1 or 2 disease or were missing grade data. Patients with histology of sarcomas and other rare subtypes were excluded (Figure S1).

### 2.4. Patient Characteristics

We reported baseline patient demographic and clinical characteristics among advanced EC patients only due to data availability. This included age, body weight, urban residence, measures of socioeconomic status (neighborhood annual household income and proportion of neighborhood with at least a high school education), the Charlson Comorbidity Index, and specific comorbidities (cardiovascular disease, diabetes, dementia, chronic obstructive pulmonary disease, connective tissue disease, paraplegia, liver disease, and renal disease). Charlson comorbidity was assessed within the 6 months prior to diagnosis using the administrative data codes described in Quan et al. (2005) [30], while body weight was measured within 60 days of diagnosis. The remaining baseline covariates were assessed at the time of diagnosis (approximately +/− one month). Patient tumor characteristics and metastatic site were also reported, including pathogenetic type (I or II), American Joint Committee on Cancer (AJCC) tumor–nodes–metastasis stage at diagnosis, the number of metastatic sites, and specific metastatic sites. Histopathological and molecular classification of EC were not available. Characteristics were stratified by those who received chemotherapy following diagnosis and those who did not.

### 2.5. Treatments and Lines of Therapy

Treatment regimens and lines of therapy were defined based on treatments received, changes in treatment regimens, and gaps in therapy, using an algorithm (Figure S2) similar to one used in a previously published study [28,31]. Specifically, the initial chemotherapy regimen was classified according to all of the chemotherapy agents received within 14 days of initiating the first line of chemotherapy. The start of the subsequent line of therapy was defined as the earliest of the following two events: receipt of any chemotherapy agent not within the initial regimen or a treatment gap of more than 90 days between successive treatment dispensations. The end date of chemotherapy was defined as the earliest of the following three possible dates: (1) the date of the last cycle of the line of therapy plus 21 days, (2) the date of starting a subsequent line of chemotherapy, or (3) the date of death or administrative censoring.

For hormone therapy, if initiated in the first line it was either classified as hormone monotherapy or a chemotherapy combination if it overlapped with the duration of first-line chemotherapy. To identify possible cases of hormone therapy in the second line, we manually checked for receipt of hormone therapy following the end of first-line therapy among individuals who did not initiate a second-line chemotherapy regimen and who did not concurrently receive hormone therapy in the first line. This process was repeated to identify third-line hormone therapy as well. The end of hormone therapy was defined as the date of the last receipt of hormone therapy or the date of death or administrative censoring, whichever was earliest. The types of therapy included in this study are shown in Table S1.

### 2.6. Outcomes

The primary study endpoints were OS and TTNT (as a proxy for disease progression). OS was defined as the time from diagnosis or from the initiation of each line of therapy until death from any cause. TTNT was defined as the time from the initiation of each line of therapy until the initiation of a new line of therapy or death from any cause, whichever came first.

### 2.7. Subgroup Analysis

A subgroup analysis was performed among patients who received PBCT in the first line to describe treatment patterns and outcomes following PBCT exposure, a treatment setting that is being targeted by emerging immunotherapies for EC, such as the PD-1 inhibitors dostarlimab and pembrolizumab [20,21].

### 2.8. Statistical Analysis

Continuous study measures were reported descriptively with mean, standard deviation, median, and interquartile range. To compare the distribution of the baseline and clinical characteristics between those who initiated chemotherapy and those who did not, *p*-values corresponding to t-tests for continuous variables and chi-square tests for categorical variables are presented. With respect to OS and TTNT, the median, two-year, and five-year survival estimates were reported along with 95% confidence intervals (95% CI). Kaplan–Meier analyses were presented overall and by line of therapy, including the type of therapy. To explore potential heterogeneity between recurrent cases and cases that initially present with advanced disease, analyses were also stratified by advanced patients versus recurrent patients. All analyses were conducted using the R computing framework (https://www.r-project.org (accessed on 15 June 2022)).

## 3. Results

### 3.1. Patient Cohort Characteristics

A total of 1053 patients were identified and included in the cohort: 620 (58.9%) with advanced-stage EC and 433 (41.1%) with recurrence following an early stage diagnosis (Table 1 and Table 2). Among advanced-stage EC patients, the mean age at diagnosis was 65 years, 56.1% had type II EC, 68.2% had a mean weight over 60 kg, and 19.0% had diabetes. Most individuals resided in an urban residence (79.7%), lived in neighborhoods with an annual household income < 45k (75.7%), and received at least a high school education (77%). Over half of the patients (59.8%) had no metastatic sites at diagnosis. Common sites of metastasis at diagnosis included the peritoneum (19.4%), pulmonary metastasis (11.8%), and lymph nodes (9.5%) (Table S2). In terms of treatments received, 72.7% of patients received chemotherapy, 76.6% received surgery, and 46.3% received radiotherapy (Table 1).

Relative to patients who did not initiate chemotherapy, patients who did receive chemotherapy were significantly younger (*p* < 0.001) and more likely to have type II EC (*p* = 0.02), to be in AJCC stage IIIC (*p* < 0.001), and to have fewer comorbidities (with Charlson comorbidity index 2+; *p* < 0.001), specifically cardiovascular disease (3.8% vs. 9.5%; *p* < 0.009). Patients who initiated chemotherapy were also less likely to have pulmonary (8.9% vs. 19.5%; *p* < 0.001), osseous (3.1% vs. 8.9%; *p* = 0.004), or hepatic metastases (2.7% vs. 7.1%; *p* = 0.02) (Table 1 and Table S2). Finally, other interventions for the primary disease differed, as patients who received chemotherapy were more likely to have had surgery (*p* < 0.001) and radiotherapy (*p* = 0.01) compared to those who did not receive chemotherapy (Table 1).

### 3.2. Treatment Patterns

**Figure 1:**
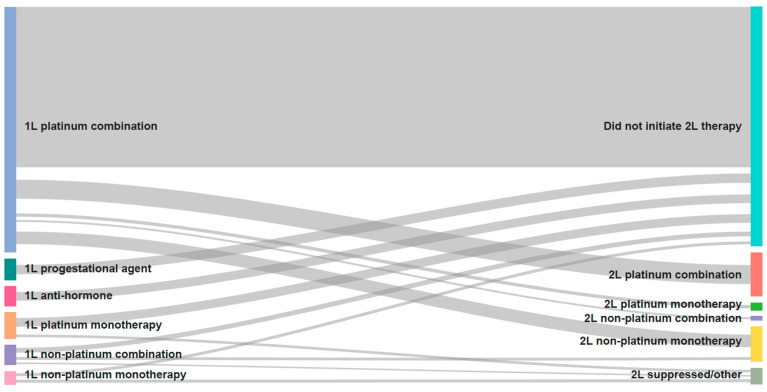
Distribution of first-line to second-line therapy across all patients. Sankey diagram showing the distribution of first-line to second-line therapy across all patients. The diagram indicates that most patients did not initiate second-line therapy.

Of the 1053 patients with advanced/recurrent EC, 713 (67.7%) initiated first-line systemic therapy (chemotherapy or hormone therapy), and this was higher among patients with advanced EC (75.2%; N = 466) compared to those with recurrent EC (57.0%; N = 247; Table 2). PBCT was the most common first-line regimen received (78.5% [N = 560]; monotherapy and combination therapy) but differed by patient type (96.1% [N = 448] for advanced patients; 45.3% [N = 112] for recurrent patients). Hormone therapy in the first line was higher for recurrent patients compared with advanced patients. The distribution of first- and second-line treatments across all patients (Sankey diagram) revealed that most patients did not initiate second-line therapy (Figure 1). A total of 257 (24.4%) patients received second-line systemic therapy, with a higher frequency in advanced versus recurrent patients (Table 2). Among the 560 patients who received PBCT in the first line, only 187 (33.4%) initiated second-line chemotherapy, 61.5% of whom were rechallenged with PBCT. Treatment differences were observed between advanced and recurrent patients in this second-line post-PBCT setting, with platinum combination therapy use higher among advanced patients and non-platinum monotherapy use higher among recurrent patients. Only 8.5% (*n* = 90) of the overall cohort received third-line therapy (Table 2).

### 3.3. Overall Survival and Time to Next Treatment

#### 3.3.1. First-Line Therapy

**Figure 2:**
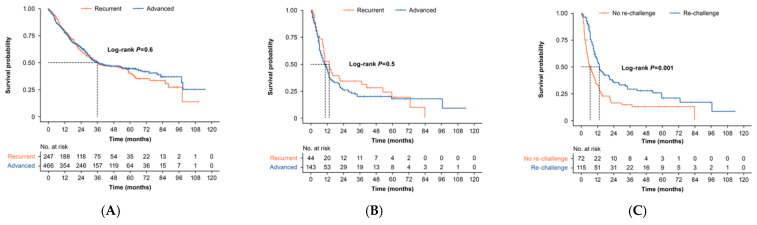
Kaplan–Meier curves of OS (unadjusted) after first-line or second-line treatment in EC patients treated with or without PBCT. (**A**) OS from first-line therapy in patients with recurrent (orange) or advanced (blue) EC. The corresponding table shows the number at risk at each time point (log-rank *p* = 0.6). (**B**) OS from second-line therapy among patients treated with first-line platinum-based chemotherapy with recurrent (orange) or advanced (blue) EC; *p* = 0.5 (**C**) OS from second-line therapy among patients who received a platinum regimen in the first line and were then rechallenged with platinum-based chemotherapy (blue) vs. those with no rechallenge (orange). Dotted lines indicate median survival at a 50% survival probability; *p* < 0.001. OS, overall survival.

The median OS from first-line therapy was 35.9 months (95% CI: 31.5–53.5) and was similar in both recurrent (35.9 months) and advanced (35.4 months) patients (Figure 2 and Table S3). Patient numbers declined after the 60-month follow-up, particularly following second-line therapy (Figure 2). TTNT from first-line therapy was 19.9 months overall (95% CI: 17.5–23.5), 18.4 months for recurrent patients, and 21.3 months for patients with advanced disease (Figure S3 and Table S4). In an unadjusted analysis, the median OS by treatment type ranged from 8.5 months (95% CI: 6.2–20.0) for platinum monotherapy to 62.5 months (95% CI: 59.2–NA) for hormone therapy (Figure S4 and Table S3).

#### 3.3.2. Second-Line Therapy

The median OS from second-line therapy (*n* = 257) was 12.6 months overall (95% CI: 10.0–14.6), 15.9 months in recurrent patients, and 10.3 months in advanced EC patients (Figure S6 and Table S3). TTNT from second-line therapy was 7.0 months overall (95% CI: 6.1–8.2), 6.4 months for recurrent patients, and 7.6 months for advanced EC patients (Figure S7 and Table S4).

For patients who received PBCT in the first-line setting, outcomes differed by treatment in the second line. The median OS was 10.4 months overall (95% CI: 8.9–13.3), 13.3 months (95% CI: 11.2–20.9) for those rechallenged with PBCT, and 6.4 months for those not rechallenged (95% CI: 4.6–10.4 months) (Table 3 and Table S3, and Figure 2). Median OS varied by the type of chemotherapy received, ranging from 6.7 months (95% CI: 4.5–11.4 months) for liposomal doxorubicin to 13.3 months (95% CI: 9.1–41.2 months) for carboplatin plus paclitaxel (Table S3 and Figure S8). Unadjusted analysis of TTNT from second-line chemotherapy following first-line PBCT was 6.4 months (95% CI: 5.3–7.7 months; Table S4 and Figure S9). Some variation in TTNT by treatment type was also observed, from 3.9 months (95% CI: 3.2–7.4 months) for liposomal doxorubicin to 9.9 months (95% CI: 7.1 months–NA) for carboplatin plus liposomal doxorubicin (Table S4 and Figure S10). Median OS from 2L was 13.4 months (95% CI: 9.3–37.4 months) in recurrent patients and 10.3 months (95% CI: 8.0–13.2 months) in advanced patients. Median TTNT from 2L was 6.5 months (95% CI: 5.2–10.0 months) in recurrent patients and 7.6 months (95% CI: 6.1–9.9 months) in advanced EC patients (Table S4 and Figure S9).

#### 3.3.3. Third-Line Therapy

As previously mentioned, only 90 (8.5%) of patients initiated third-line treatment. The median OS for third-line therapy was 11.0 months overall (95% CI: 8.2–13.5 months), 12.0 months (95% CI: 8.0–27.6) in recurrent patients, and 11.0 months (95% CI: 8.1–15.8) in patients with advanced EC. Median TTNT was 7.2 months overall (95% CI: 6.0–10.2 months), 9.0 months (95% CI: 6.4–12.2) in recurrent patients (N = 41), and 5.3 months (95% CI: 4.7–10.5) in patients with advanced EC (N = 49) (Tables S3 and S4, Figures S11 and S12).

## 4. Discussion

The aim of this retrospective, population-based study was to describe treatment patterns and examine real-world outcomes by line of therapy for patients with advanced and recurrent EC in Alberta, Canada. As far as we are aware, this is the first report of real-world data for this patient population in Canada. Our study demonstrates that treatment options for patients with advanced/recurrent EC are limited, with a high proportion receiving no systemic treatment following their advanced-stage diagnosis or their recurrence following early stage diagnosis (25% of advanced stage patients and 43% of recurrent patients), highlighting an unmet need for treatment options for these patients. Since this is an administrative study, we cannot discern the true reason for the high proportion of patients who received no systemic treatments; however, this may be as a result of patient preference, patient fitness, age, comorbidities, the extent of disease, or other unmeasured factors. Additionally, some patients may have received treatment with only surgery and/or radiation. Alberta has a publicly funded health system, and patients with advanced or recurrent EC are referred to primary care centers where a treatment plan is discussed by a tumor board, and treatment is primarily provided by a gynecologic oncologist. Our study also shows that currently available treatment options are associated with poor outcomes, particularly from second-line therapy and onwards. For patients who received first-line therapy in our study, PBCT was used for most (96.2%) patients with advanced-stage disease and nearly half of patients with recurrent disease (45.4%), which is consistent with the current treatment guidelines and previously published data [6,10,15,32]. Single-agent chemotherapy, which has been well evaluated in the relapsed clinical setting [33,34,35], was used increasingly from the second line onwards in our study. An analysis conducted on patients with EC (stage I–IV) in Japan had similar findings suggesting that these patterns of treatment are not unique to Canada [32]. The observation that most patients did not initiate second-line therapy (76%) may be explained by the limited efficacy and the toxicity associated with standard treatments [11,36]. End-of-life care planning is an option provided to patients in this setting [9].

Poor survival outcomes were observed following both first- and second-line treatment. Median OS and TTNT from first-line systemic therapy were 35.9 months and 19.9 months, respectively, while the median OS from second-line chemotherapy was only 12.6 months (10.4 months among patients who received first-line PBCT). TTNT from the second line overall was also much lower than the first line at just 7.0 months (95% CI: 6.1–8.2). These real-world outcomes observed following second-line chemotherapy are similar to those reported in previous studies [25,34,37,38]. Notably, a recent, real-world study of patients with advanced/recurrent EC (*n* = 999) in England reported similar OS and TTNT following second-line treatment to those reported in our study [38]. Rechallenge with PBCT is an option for patients who relapse following first-line therapy [6,7,29]. In our study, just over half of the 24% of patients who initiated second-line treatment were rechallenged with PBCT. These patients appeared to have better survival (13.3 months with PBCT rechallenge and 6.4 months with no rechallenge), which is likely reflective of both the relative efficacy of this treatment modality and the overall fitness of the patients. Furthermore, while PBCT rechallenge was associated with the best outcomes of any chemotherapy utilized, survival outcomes were still poor and aligned to clinical trials in this setting where a median OS of 10.3 months with paclitaxel rechallenge and up to 15 months with carboplatin and paclitaxel rechallenge have been reported [36,39].

Several therapeutic treatment regimens were used by patients in this real-world study with some significant differences in patient outcomes (OS and TTNT) by treatment type observed across treatment lines. No comparative efficacy statements can be made on the basis of these descriptive results because we did not control for confounding factors (such as patient characteristics) and other potential sources of bias, which should be explored in future studies. However, these observations clearly highlight the absence of a single standard of care following first-line treatment and the poor outcomes for these patients regardless of intervention.

There is limited guidance for the treatment of patients with advanced/recurrent EC in Canada, with current guidelines strongly suggesting enrolment in clinical trials [6,9]. The poor outcomes observed in this study highlight the urgent need for novel therapies that can improve outcomes for patients with advanced and recurrent EC. Indeed, ongoing trials are investigating new treatments to focus on select types of EC and to personalize treatment strategies [16,17]. In recent years, immune checkpoint inhibitors have demonstrated efficacy and manageable safety profiles in patients with biomarker-selected advanced/recurrent EC and could improve survival outcomes in the post-platinum setting [23,25]; however, access to these treatments remains limited in Canada. In addition to the second-line setting, there is also significant interest in immunotherapy as a potential treatment option in the first-line setting for EC, either as monotherapy or in combination with other agents. Several clinical trials are ongoing, which may provide further treatment options for women with advanced/recurrent EC [40,41,42,43,44,45].

This study has several strengths and limitations which should be noted. A key strength is the population-based cohort study design, which captures all individuals with a diagnosis of EC. The databases leveraged in this analysis capture detailed information on patient characteristics, diagnoses, procedures, and treatments received over their lifetime, facilitating the assessment of treatment patterns and patient outcomes. The ability to link diverse provincial databases provides a comprehensive source of information for health outcomes studies. However, one limitation is that the provincial cancer registry only captures detailed information at initial cancer diagnosis. As such, demographic information that changes over time may be missing, particularly in patients with recurrent EC. In addition, certain diagnostic variables such as EC molecular classification data (i.e., MMR or MSI status) were not available; therefore, outcomes within subgroups of patients who are either MMR deficient or proficient are unknown. However, this would be valuable to examine in future studies where these variables are available. Stage III disease may present as either microscopic lymph node metastasis, macroscopic lymph node metastasis, or adnexal involvement, all of which may have different treatment approaches or survival outcomes [46]. These factors are increasingly important as the diagnosis and management of EC evolves towards personalized medicine, where specific treatments are tailored towards the individual histology, molecular characteristics, and stage of the tumor at diagnosis [16,17]. In this study, we were able to look at stage IIIB versus stage IIIC disease (stage IIIA was excluded from this cohort), but any further granularity was not available. The recurrence status in this patient population was estimated by an algorithm. Similar approaches have been used in previous studies [28,31,47]; however, some patients may have been misclassified as recurrent due to algorithm errors. Additionally, an algorithm was implemented to define lines of therapy based on changes in treatment regimens received and gaps in therapy. This may have led to misclassifications if changes in treatments were part of the same line of therapy. However, we expect the risk of this to be very low based on current treatment practices outlined in the guidelines [7]. It should also be noted that the use of algorithms is a limitation of real-world studies generally and is not unique to this study [31,34]. Due to a lack of HC approval during the study period, the use of novel PD-1 inhibitors was not captured in this study. Lastly, patient factors influencing treatment decisions are unknown in this study.

## 5. Conclusions

Treatment options are limited for patients with advanced/recurrent EC in Alberta, Canada. Many patients in this setting go without treatment. For patients who do receive treatment, real-world survival rates are poor, particularly in the second-line treatment setting following disease progression with existing chemotherapy and hormonal therapy options. A lack of effective and well-tolerated regimens represents an area of unmet need for patients in Canada. Novel agents, such as immunotherapies with proven efficacy, have the potential to improve EC outcomes for biomarker-selected patients, although timely access to these emerging therapies through public payer or private systems is urgently needed.

## Figures and Tables

**Table 1:** Baseline and clinical demographics for advanced patients with EC, by receipt of chemotherapy.

Variable	Overall (*n* = 620)	Chemo ^1^ (*n* = 451)	No Chemo ^2^ (*n* = 169)	*p*-Value
**Age, years (mean ± SD, range)**	65 ± 11 31–98	64 ± 10 31–92	69 ± 13 35–98	<0.001 ^3^
<60 years, *n* (%)	185 (29.8)	145 (32.2)	40 (23.7)	<0.001 ^4^
60–<70 years, *n* (%)	224 (36.1)	181 (40.1)	43 (25.4)	––
70+, *n* (%)	211 (34.0)	125 (27.7)	86 (50.9)	––
**Weight, kg (mean ± SD) ^5^**	81.4 ± 23.1	81.7 ± 22.6	80.5 ± 24.8	0.66 ^3^
<60 kg, *n* (%)	82 (13.2)	64 (14.2)	18 (10.6)	0.89 ^4^
60+ kg, *n* (%)	423 (68.2)	336 (74.5)	87 (51.5)	––
Missing information, *n* (%)	115 (18.5)	51 (11.3)	64 (37.9)	––
**Charlson Comorbidity Index, *n* (%)**				<0.001 ^4^
0	433 (69.8)	333 (73.8)	100 (59.2)	––
1	118 (19.0)	81 (18.0)	37 (21.9)	––
2+	69 (11.1)	37 (8.2)	32 (18.9)	––
**Tumor characteristics and metastatic sites, *n* (%)**				
**Type**				0.02 ^4^
I	272 (43.9)	184 (40.8)	88 (52.1)	––
II	348 (56.1)	267 (59.2)	81 (47.9)	––
**AJCC Stage**				<0.001 ^4^
IIIB	42 (6.8)	19 (4.2)	23 (13.6)	––
IIIC	329 (53.1)	263 (58.3)	66 (39.1)	––
IV	249 (40.2)	169 (37.5)	80 (47.3)	––
**Number of metastatic sites at diagnosis**				0.11 ^4^
0	371 (59.8)	282 (62.5)	89 (52.7)	––
1	149 (24.0)	106 (23.5)	43 (25.4)	––
2	54 (8.7)	36 (8.0)	18 (10.7)	––
3+	35 (5.6)	21 (4.7)	14 (8.3)	––
Missing	11 (1.8)	<10 (<2.2)	<10 (<5.9)	––
**Treatment characteristics, *n* (%)**				
**Surgery**	475 (76.6)	382 (84.7)	93 (55.0)	<0.001 ^4^
**Radiotherapy**	287 (46.3)	224 (49.7)	63 (37.3)	0.01 ^4^

Due to privacy regulations, cell counts <10 cannot be disclosed. Individual percentage values are rounded and may not total 100%. ^1^ *n* < 10 did not receive platinum chemotherapy; ^2^ 15 patients received hormone therapy in the first line and all other patients did not receive hormone therapy; ^3^ *p*-value corresponding to t-test; ^4^ *p*-value corresponding chi-square test; ^5^ Measure taken within +/− 60 days of diagnosis that was closest to the date of diagnosis. AJCC, American Joint Committee on Cancer; Chemo, chemotherapy; EC, endometrial cancer; SD, standard deviation.

**Table 2:** Treatment patterns for patients with advanced and recurrent EC.

Patients Who Received:	Overall	Advanced EC	Recurrent EC
	(N = 1053)	(N = 620; 58.9%)	(N = 433; 41.1%)
No systemic therapy	N = 340 (32.3%)	N = 154 (24.8%)	N = 186 (43.0%)
**1L systemic therapy, *n* (%)**	N = 713 (67.7%)	N = 466 (75.2%)	N = 247 (57.0%)
Platinum combination	506 (71.0)	410 (88.0)	96 (38.9)
Platinum monotherapy	54 (7.6)	38 (8.2)	16 (6.5)
Non-platinum combination	41 (5.8)	<10 (<2.1)	66 (26.7)
Non-platinum monotherapy	28 (3.9)		
Progestational agent/hormone therapy	84 (11.8)	15 (3.2)	69 (27.9)
**2L systemic therapy, *n* (%)**	N = 257 (24.4%)	N = 169 (27.3%)	N = 88 (20.32%)
Platinum combination	97 (37.7)	79 (46.7)	18 (20.5)
Platinum monotherapy	20 (7.8)	>10 (>5.9)	<10 (<11.4)
Non-platinum combination	18 (7.0)	<10 (<5.9)	>8 (>9.1)
Non-platinum monotherapy	85 (33.1)	45 (26.6)	40 (45.5)
Progestational agent/hormone therapy	37 (14.4)	25 (14.8)	12 (13.6)
**2L systemic therapy following 1L PBCT, *n* (%)**	N = 187 (17.8%)	N = 144 (23.2%)	N = 43 (9.9)
Platinum combination	96 (51.3)	79 (54.9)	17 (39.5)
Platinum monotherapy	19 (10.2)	>9 (6.3)	<10 (<23.0)
Non-platinum combination	<10 (<5.3)	<10 (<6.9)	<10 (<23.0)
Non-platinum monotherapy	65 (34.8)	44 (30.6)	21 (48.8)
**3L systemic therapy, *n* (%)**	N = 90 (8.5%)	N = 39–57 (<9.2%)	N = 33–41 (<9.5%)
Non-platinum monotherapy	40 (44.4)	22	18
Platinum combination	22 (24.4)	>13	<10
Other chemotherapy	<10 (<11.1)	<10	<10
Progestational agent/hormone therapy	19 (21.1)	>10	<10

Due to privacy regulations, cell counts <10 cannot be disclosed. Individual percentage values are rounded and may not total 100%. 1L, first line; 2L, second line; 3L, third line; EC, endometrial cancer; PBCT, platinum-based chemotherapy.

**Table 3:** OS after first- or second-line treatment in EC patients.

		Median Survival, Months (95% CI)	2-Year Survival Probability (95% CI)	5-Year Survival Probability (95% CI)
**OS from 1L, by disease type**	All (N = 713)	35.9 (31.5–53.5)	0.63 (0.59–0.67)	0.43 (0.39–0.47)
	Recurrent (N = 247)	35.9 (29.0–58.9)	0.61 (0.55–0.68)	0.40 (0.33–0.49)
	Advanced (N = 466)	35.4 (30.9–57.5)	0.64 (0.60–0.69)	0.44 (0.39–0.50)
**OS from 2L among patients treated with 1L PBCT, by disease**	All (N = 187)	10.4 (8.9–13.3)	0.28 (0.22–0.35)	0.17 (0.12–0.26)
	Recurrent (N = 43)	13.4 (9.3–37.4)	0.34 (0.22–0.53)	0.19 (0.09–0.40)
	Advanced (N = 144)	10.3 (8.0–13.2)	0.26 (0.19–0.35)	0.18 (0.11–0.27)
OS from 2L among patients treated with 1L PBCT, by therapy *	All (N = 187)	10.4 (8.9–13.3)	0.28 (0.22–0.35)	0.17 (0.12–0.26)
	Rechallenged (N = 115)	13.3 (11.2–20.9)	0.35 (0.27–0.46)	0.21 (0.13–0.33)
	Not Rechallenged (N = 72)	6.4 (4.6–10.4)	0.16 (0.09–0.28)	0.13 (0.07–0.24)

* *p* < 0.001. CI, confidence interval.

## Data Availability

Data that support the findings reported in this study are openly available or upon request from Alberta Cancer Registry at: https://public.tableau.com/app/profile/cancercontrol.ab/viz/The2021ReportonCancerStatisticsinAlberta/Highlights (accessed on 15 June 2022)), the Alberta population registry (url: https://www.cihi.ca/en/access-data-and-reports (accessed on 15 June 2022)), the discharge abstract, National Ambulatory Care Reporting System, practitioner claims (url: https://www.cihi.ca/en/national-physician-database-metadata (accessed on 15 June 2022)), and statistics Canada (url: https://www150.statcan.gc.ca/n1/en/type/data?MM=1 (accessed on 15 June 2022)) databases.

## Supplementary Materials

The following supporting information can be downloaded at: https://www.mdpi.com/article/10.3390/curroncol30020176/s1. Figure S1: Advanced and recurrent EC algorithm; Figure S2: Algorithm for classifying lines of therapy and regimens; Figure S3: TTNT from first-line therapy presented overall (A) and by disease type (B); Figure S4: OS from first-line therapy presented overall (A) and by treatment (B); Figure S5: TTNT from first-line therapy presented by treatment; Figure S6: OS from second-line therapy presented overall (A) and by disease type (B); Figure S7: TTNT from second-line therapy presented overall (A) and by disease type (B); Figure S8: Time from second-line therapy among patients treated with a platinum regimen in the first line by treatment type; Figure S9: TTNT from second-line therapy among patients treated with first-line PBCT presented overall (A) and by disease type (B); Figure S10: TTNT from second-line therapy among patients treated with first-line PBCT presented by treatment (A) and drug (B); Figure S11: OS from third-line therapy presented overall (A) and by disease type (B); Figure S12: TTNT from third-line therapy presented overall (A) and by disease type (B); Table S1: Additional baseline and clinical demographics for advanced EC by receipt of chemotherapy; Table S2: Overall survival by line of therapy by patient type and therapy type; Table S3: TTNT by line of therapy, patient type, and treatment type; Table S4: TTNT by line of therapy, patient type, and treatment type.
