# Supplementary material for: Understanding Characteristics, Treatment Patterns, and Clinical Outcomes for Individuals with Advanced or Recurrent Endometrial Cancer in Alberta, Canada: A Retrospective, Population-Based Cohort Study
Source: Curr Oncol. 2023 Feb 14;30(2):2277–89. doi: 10.3390/curroncol30020176 (PMC9955469; doi:10.3390/curroncol30020176)
Supplement: Supplementary file 1 [file curroncol-30-00176-s001.zip › curroncol-2195128-supplementary.pdf]

Current Oncology

MDPI

Supplementary Materials

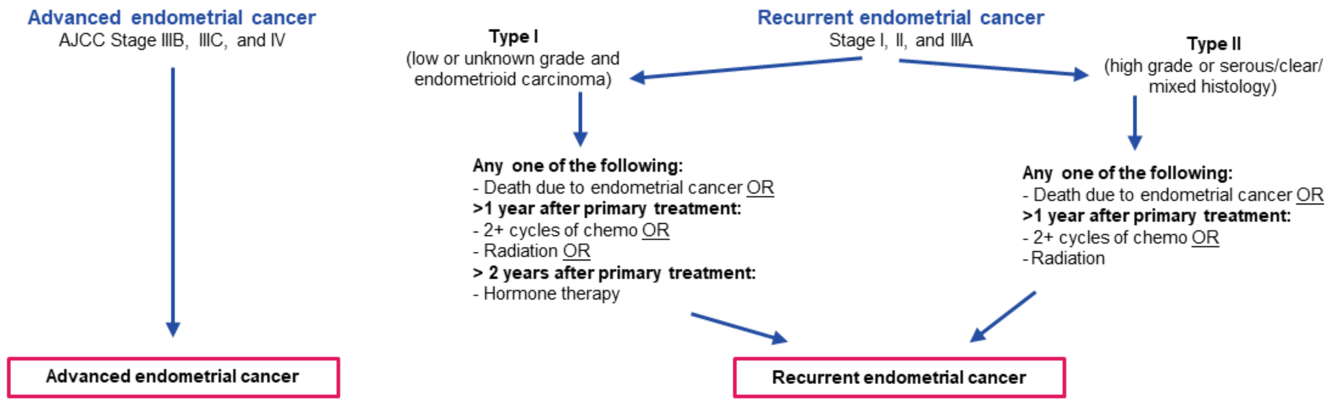

Figure S1. Advanced and recurrent EC algorithm.

AJCC, American Joint Committee on Cancer; EC, endometrial cancer.

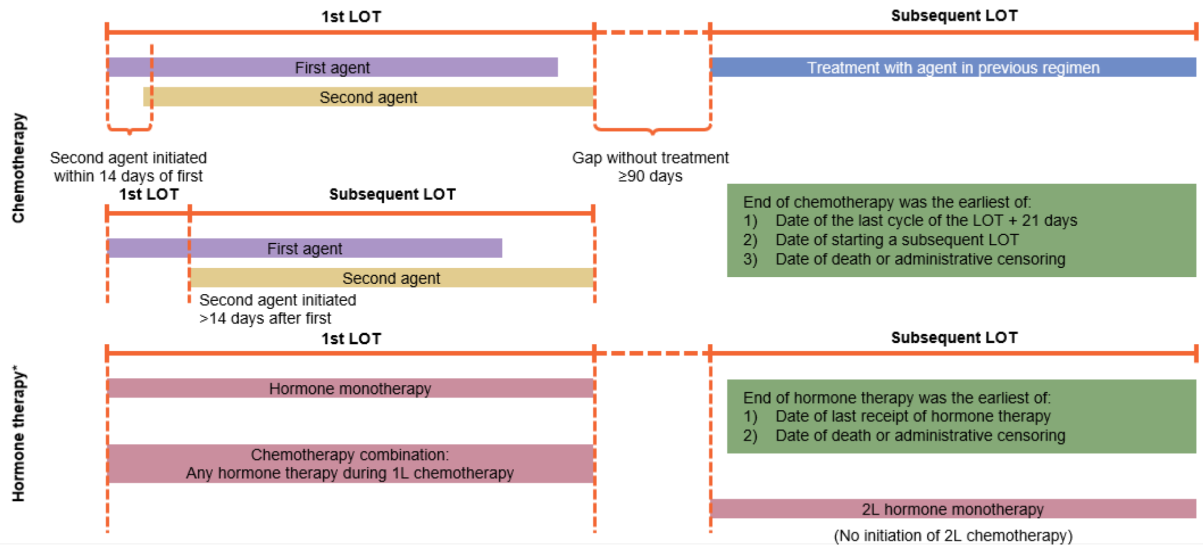

Figure S2. Algorithm for classifying lines of therapy and regimens.

*If hormone therapy was initiated in 1L it was either classified as a hormone monotherapy or a chemotherapy combination. 1L, first-line; 2L, second-line; LOT, line of therapy.

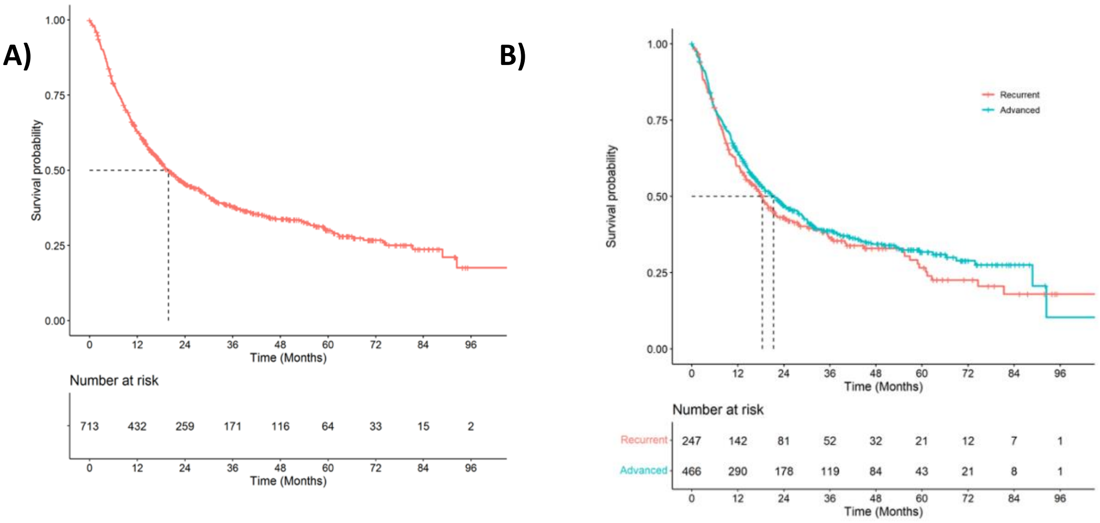

Figure S3. Unadjusted TTNT from first-line presented overall (A) and by disease type (B).

TTNT from first-line treatment in the overall cohort **(A)** and by disease type (unadjusted); $p = 0.3$. **(B)** The corresponding data tables show the number of patients at risk at each time point. The dotted lines depict the median survival at the 50% survival probability. TTNT, time to next treatment.

A)

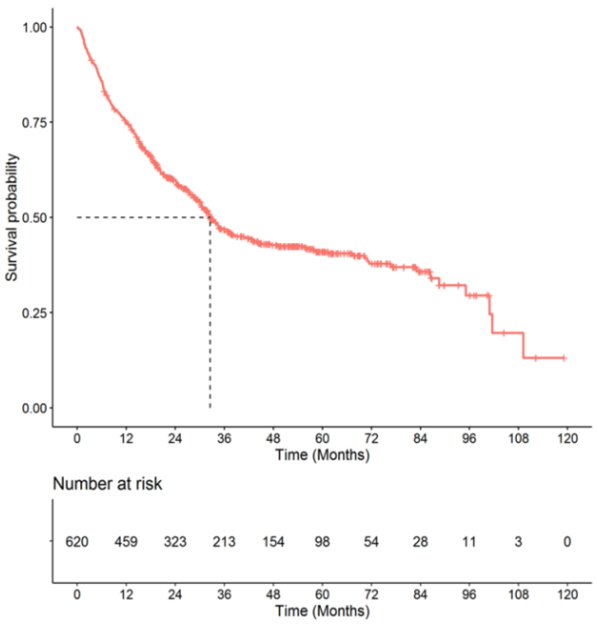

B)

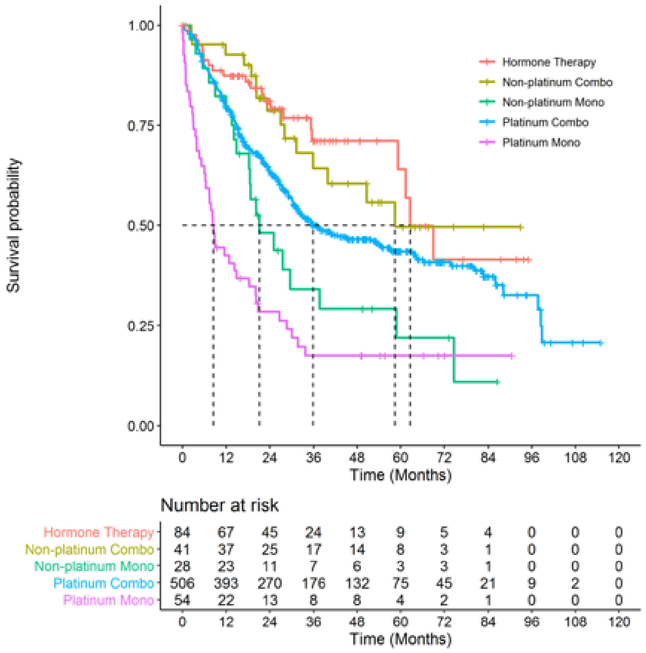

Figure S4. Unadjusted OS from first-line, presented overall (A) and by treatment (B).

(A) OS (months) of the entire cohort. **(B)** OS (unadjusted; months) stratified by first-line therapy (hormone therapy, non-platinum chemotherapy combination, non-platinum chemotherapy monotherapy, platinum chemotherapy combination, or platinum chemotherapy monotherapy); $p < 0.001$. The corresponding data tables show the number of patients at risk at each time point. The dotted lines indicate the median survival at 50% survival probability. Combo, combination therapy; mono, monotherapy.

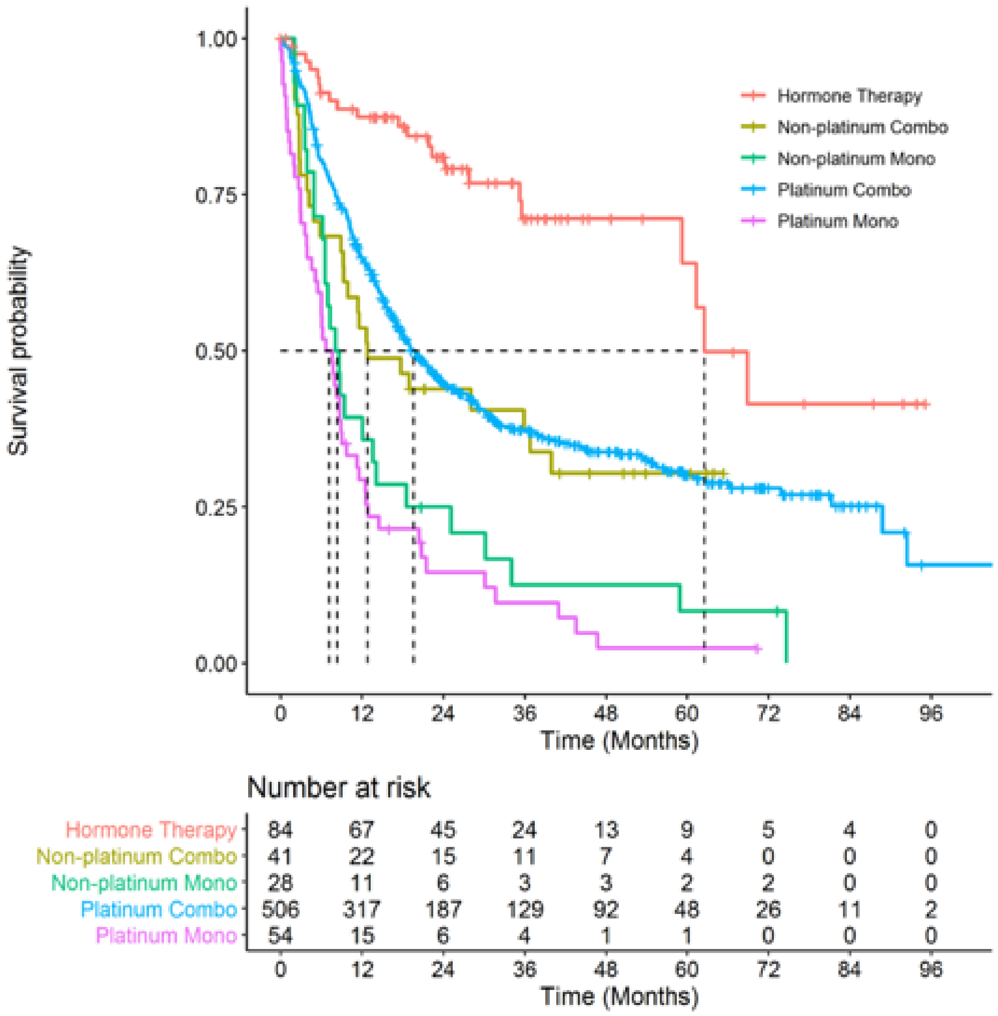

Figure S5. Unadjusted TTNT from first-line presented by treatment**.**

TTNT (unadjusted) following first-line therapy by treatment type; $p < 0.001$. The corresponding data tables show the number of patients at risk at each time point. The dotted line depicts median survival at the 50% survival probability.

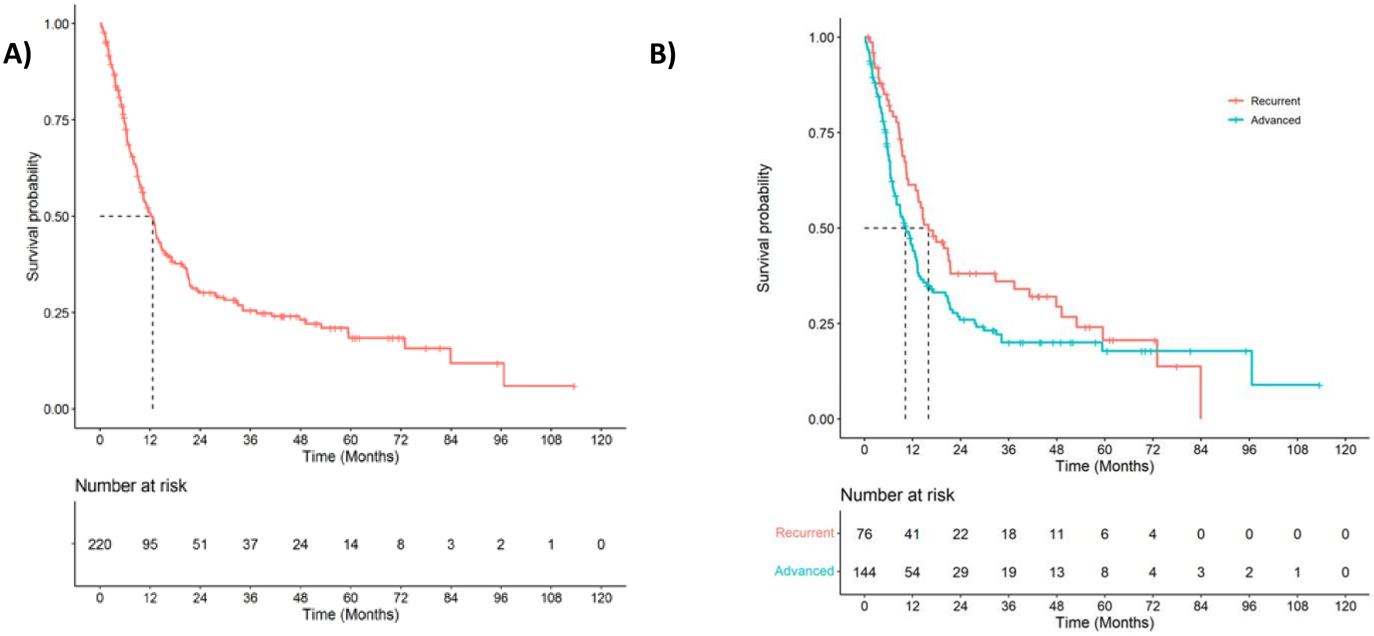

Figure S6. Unadjusted OS from second-line, presented overall (A) and by disease type (B).

(A) OS (in months) of the entire cohort. **(B)** OS (in months; unadjusted) by recurrent (shown in red) or advanced (shown in blue) EC; $p = 0.06$. The corresponding data tables show the number of patients at risk at each time point. The dotted line depicts median survival at the 50% survival probability.

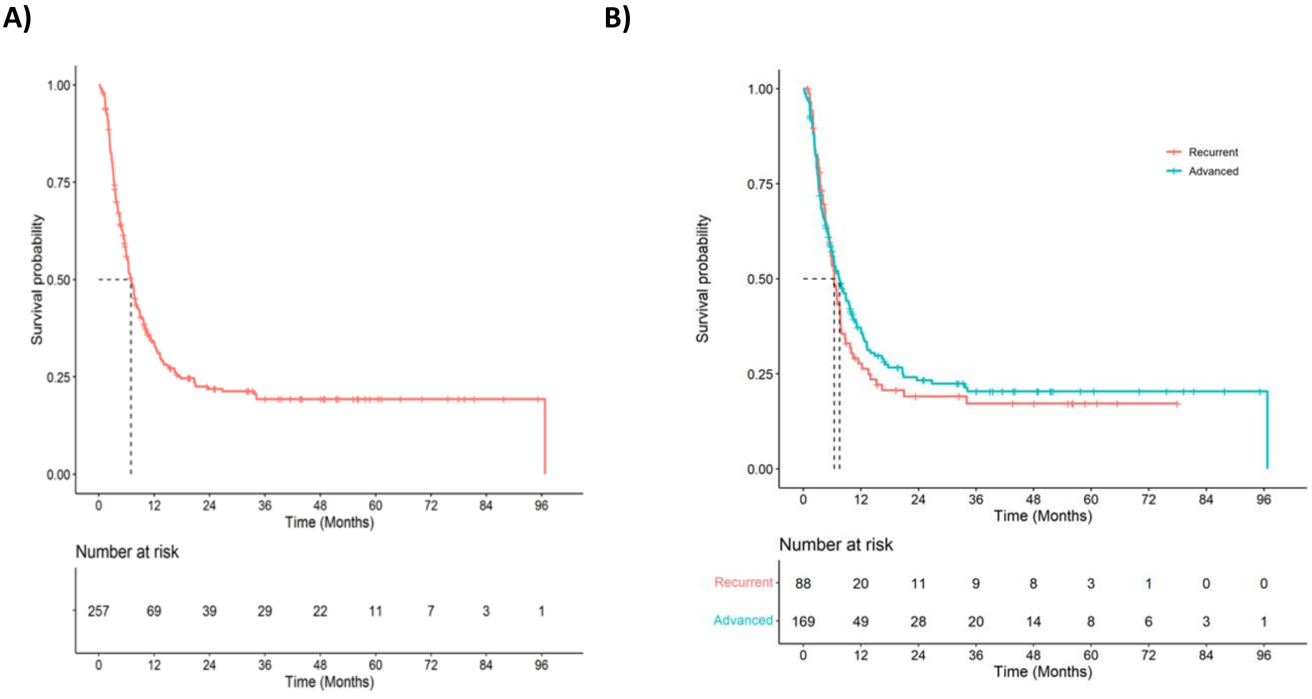

Figure S7. Unadjusted TTNT from second-line, presented overall (A) and by disease type (B).

TTNT following second-line therapy presented as the overall cohort; $p = 0.4$ **(A)** and by disease type (unadjusted) **(B)**. The corresponding data tables show the number of patients at risk. The dotted line depicts median survival at 50% survival probability.

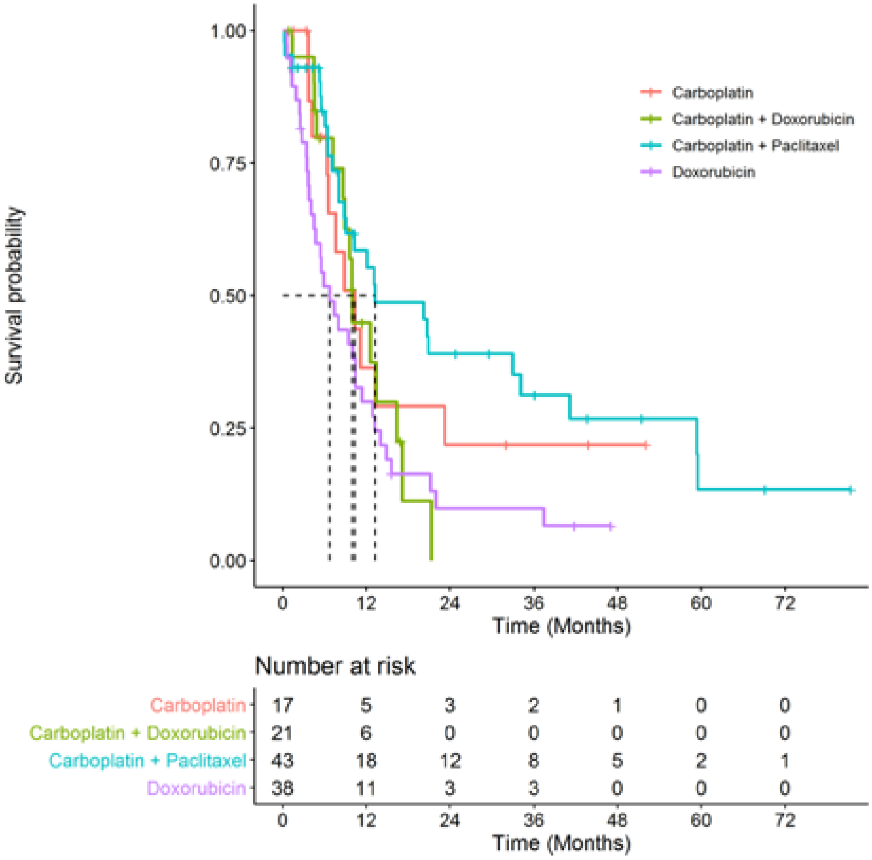

Figure S8. Time from second-line among patients treated with a platinum regimen in first-line, by treatment type.

OS (unadjusted) in patients treated with first-line PBCT treatment type. The corresponding data tables show the number of patients at risk at each time point; $p = 0.02$. The dotted lines depict the median survival at the 50% survival probability.

A)

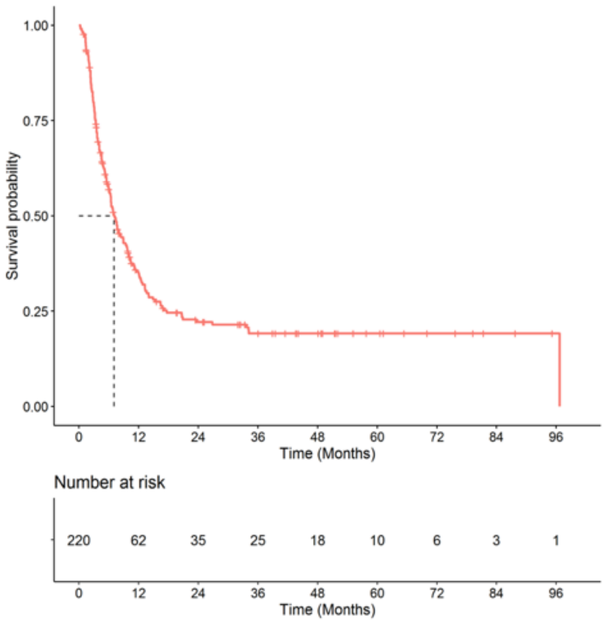

B)

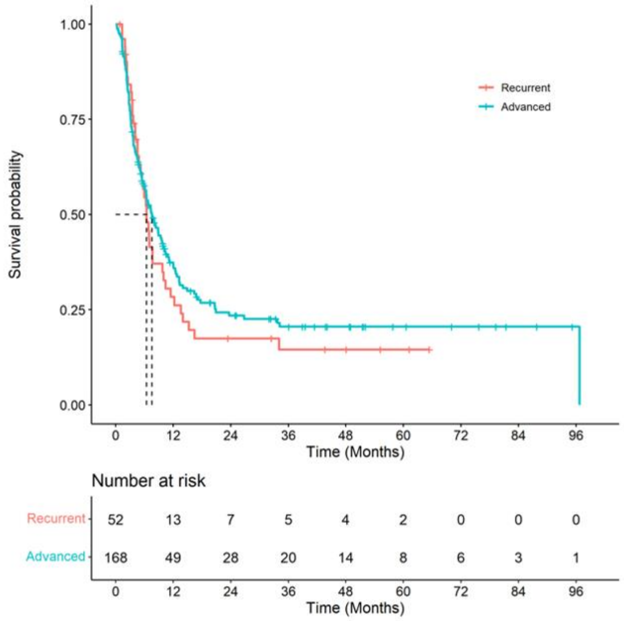

Figure S9. Unadjusted TTNT from second-line among patients treated with first-line PBCT, presented overall (A) and by disease type (B).

TTNT following second-line therapy among patients treated with PBCT as first-line therapy by the overall cohort; $p = 0.5$ **(A)** and by disease type (unadjusted) **(B)**. The corresponding data tables show the number of patients at risk. The dotted lines depict the median survival at 50% survival probability.

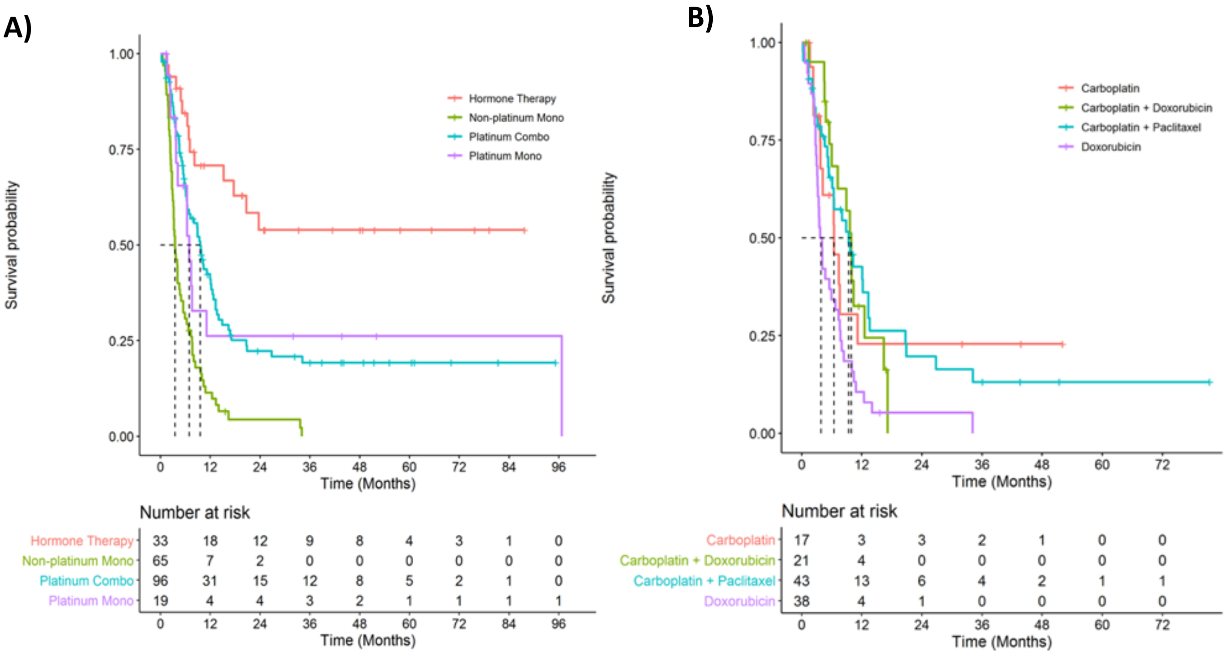

Figure S10. Unadjusted TTNT from second-line among patients treated with first-line PBCT, presented by treatment (A) and drug (B).

TTNT following second-line treatment among patients treated with as first-line treatment by treatment type (unadjusted); $p < 0.001$ (A) and specific drugs (unadjusted); $p = 0.003$ (B). The corresponding data tables show the number of patients at risk. The dotted lines depict median survival at the 50% survival probability.

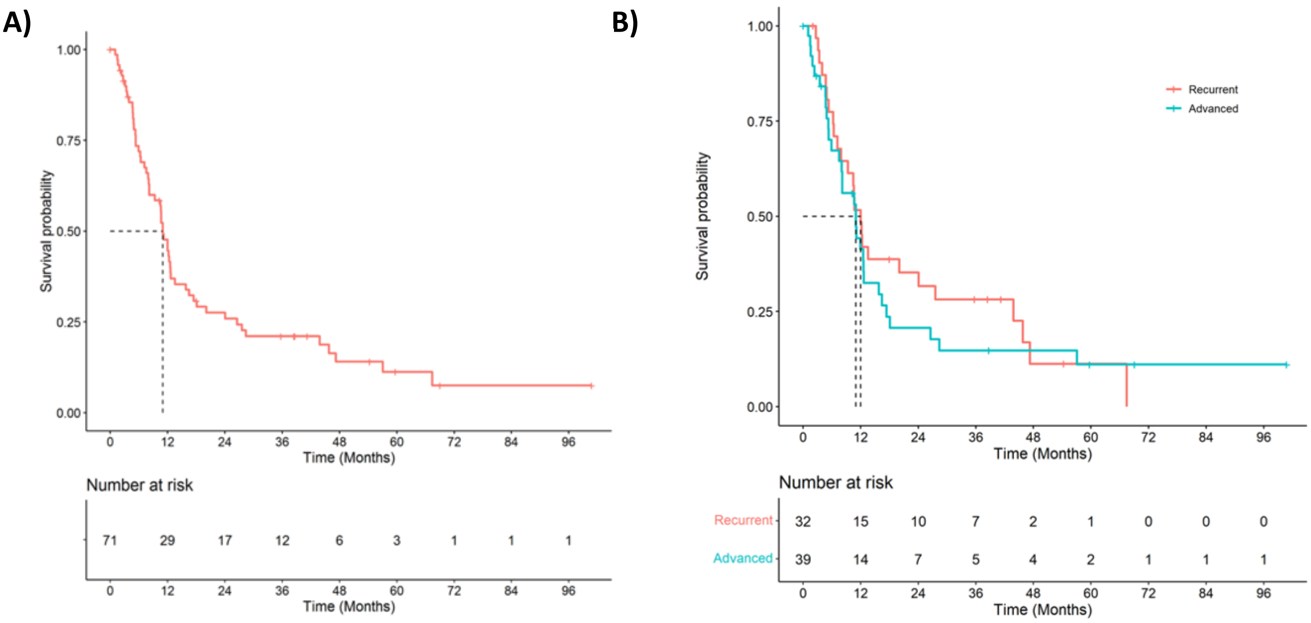

Figure S11. Unadjusted OS from third-line presented overall (A) and by disease type (B).

OS following third-line treatment in patients who received PBCT as first-line treatment overall **(A)** and by disease type (unadjusted); p = 0.6 **(B)**. The corresponding data tables show the number of patients at risk at each time point. The dotted lines depict median survival at 50% survival probability.

A)

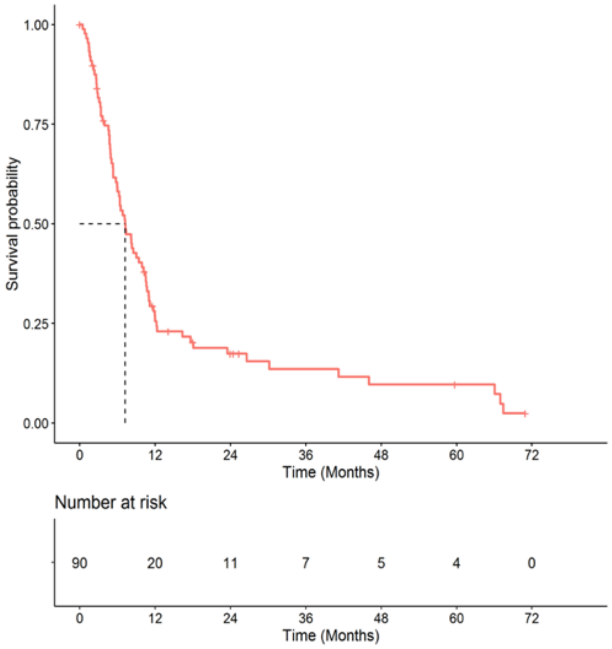

B)

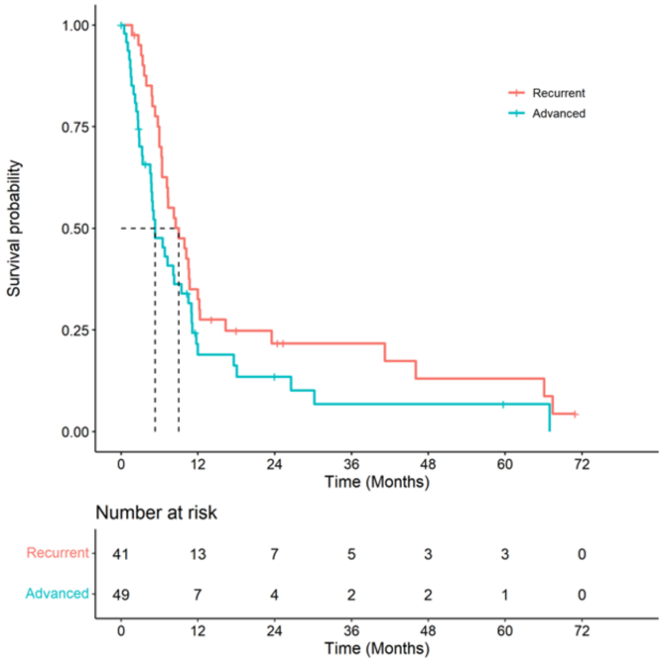

Figure S12. Unadjusted TTNT from third-line presented overall (A) and by disease type (B).

TTNT following third-line therapy presented by the overall cohort (A) and by disease type (unadjusted); *p* = 0.06 (B). Log-rank test revealed no significant difference in TTNT following third-line therapy by disease type. The corresponding data tables show the number of patients at risk. The dotted line depicts median survival at the 50% survival probability.

Table S1. Types of therapy in first, second and third-line treatment.

Therapy	First-line (N = 713)	Second-line (N = 220)	Third-line (N = 62)
**Platinum combination**	**N = 506 (71.0%)**	**N = 97 (44.1%)**	**N = 22 (35.5%)**
Carboplatin + paclitaxel	468 (92.5)	41 (42.3)	<10 (< 45.5)
Carboplatin + doxorubicin	<10 (< 2.0)	21 (21.7)	<10 (< 45.5)
Carboplatin + other	30 (5.9)	17 (17.5)	
Cisplatin + other	-	-	14 (63.6)
Cisplatin combination	<10 (< 2.0)	18 (18.6)	<10 (< 45.5)
**Platinum monotherapy**	**N = 54 (7.6%)**	**N = 20 (9.1%)**	**-**
Carboplatin	>44 (> 81.5)	>10 (> 50.0)	-
Cisplatin	<10 (< 18.5)	<10 (< 50.0)	-
**Non-platinum combination***	**N = 41 (5.8%)**	**N = 18 (8.2%)**	**-**
**Non-platinum monotherapy**	**N = 28 (3.9%)**	**N = 85 (38.6%)**	**N = 40 (64.5%)**
Doxorubicin	<10 (< 35.7)	46 (54.1)	10 (25.0
Topotecan	-	-	11 (27.5)
Other therapies†	All <10 (< 35.7)	39 (45.9)	19 (47.5)
**Progestational agent**	**N = 44 (6.2%)**	**-**	**-**
Medroxyprogesterone acetate	25 (56.8)	-	-
Megestrol acetate	19 (43.2)	-	-
**Anti-hormone therapy**	**N = 40 (5.6%)**	**-**	**-**
Letrozole	16 (40.0)	-	-
Tamoxifen	14 (35.0)	-	-
Anastrozole	10 (25.0)	-	-

Individual percentage values are rounded and may not total 100%. Due to privacy regulations, cell counts <10 cannot be disclosed. *All specific regimens included <10 patients; †All monotherapies other than doxorubicin and topotecan. All specific therapies included <10 patients.

Table S2. Additional baseline and clinical demographics for advanced EC, by receipt of chemotherapy.

Variable	Overall (N = 620)	Chemo[1] (N = 451)	No Chemo[2] (N = 169)	*P*-Value[3]
**Co-existing conditions**				
**Cardiovascular disease (%)**[4]	33 (5.3)	17 (3.8)	16 (9.5)	0.009
**Diabetes (%)**	110 (19.0)	86 (19.1)	42 (24.9)	0.14
**Dementia (%)**	11 (1.8)	<10	<10	—
**Chronic obstructive pulmonary disease (%)**	32 (5.2)	20 (4.4)	12 (7.1)	0.26
**Connective tissue disease (%)**	<10	<10	<10	—
**Paraplegia (%)**	<10	<10	<10	—
**Liver disease (%)**	<10	<10	<10	—
**Renal disease (%)**	13 (2.1)	<10	<10	—
**Tumor characteristics and metastatic sites**				
**Site of metastasis at diagnosis (%)**				
**Peritoneum**	120 (19.4)	95 (21.1)	25 (14.8)	0.1
**Pulmonary**	73 (11.8)	40 (8.9)	33 (19.5)	<0.001
**Lymph nodes**	59 (9.5)	38 (8.4)	21 (12.4)	0.18
**Osseous**	29 (4.7)	14 (3.1)	15 (8.9)	0.004
**Hepatic**	24 (4.2)	12 (2.7)	12 (7.1)	0.02
**Pleura**	24 (4.2)	20 (4.4)	<10	0.34
**Other**	51 (8.2)	32 (7.1)	19 (11.2)	0.1
**Urban residence (%)**	494 (79.7)	365 (80.9)	129 (76.3)	0.25
**Categories neighborhood annual household income (%)**				0.44
**0- < 25k**	37 (6.0)	25 (5.5)	12 (7.1)	—
**25- < 35k**	241 (38.9)	169 (37.5)	72 (42.6)	—
**35- < 45**	191 (30.8)	141 (31.3)	50 (29.6)	—
**45k+**	151 (24.3)	116 (25.7)	35 (20.7)	—
**Proportion of neighborhood residents who achieved a high school education or greater (mean [SD])**	0.77 (0.11)	0.77 (0.10)	0.76 (0.11)	0.24
**Categories of neighborhood education (%)**[5]				0.79
**0.00- < 0.60**	49 (7.9)	33 (7.3)	16 (9.5)	—
**0.60- < 0.70**	108 (17.4)	79 (17.5)	29 (17.2)	—
**0.70- < 0.80**	200 (32.3)	144 (31.9)	56 (33.1)	—
**0.80+**	263 (42.4)	195 (43.2)	68 (40.2)	—

Due to privacy regulations, cell counts <10 cannot be disclosed. [1]n < 10 did not receive platinum chemotherapy. [2]15 patients received hormone therapy in first-line, all other patients did not receive hormone therapy. [3]*p*-value corresponding to chi-square test. [4]Includes congestive heart failure, myocardial infarction, peripheral vascular disease, and cerebrovascular disease (assessed within 6 months of diagnosis). [5]Neighborhood education categories were taken from census data and are linked to postal code. Chemo, chemotherapy; SD, standard deviation.

Table S3. Overall survival by line of therapy, patient type, and therapy type.

Strata	Median Survival (95% CI)	2-Year Survival (95% CI)	5-Year Survival (95% CI)	Log Rank Test *p*-value[1]
**Overall survival from time of 1L treatment**				
**Overall**	35.9 (31.5–53.5)	0.63 (0.59–0.67)	0.43 (0.39–0.47)	—
**Patient**				$p = 0.6$
**Recurrent**	35.9 (29.0–58.9)	0.61 (0.55–0.68)	0.40 (0.33–0.49)	—
**Advanced**	35.4 (30.9–57.5)	0.64 (0.60–0.69)	0.44 (0.39–0.50)	—
**Treatment type**				$p = 0.001$
**Platinum combination**	35.9 (31.3–54.7)	0.64 (0.59–0.68)	0.44 (0.39–0.49)	—
**Platinum monotherapy**	8.5 (6.2–20.0)	0.28 (0.18–0.44)	0.18 (0.10–0.32)	—
**Non-platinum monotherapy**	21.1 (18.3–NA)	0.79 (0.66–0.93)	0.50 (0.33–0.74)	—
**Non-platinum combination**	58.4 (35.9–NA)	0.48 (0.32–0.72)	0.22 (0.09–0.51)	—
**Hormone therapy**	62.5 (59.2–NA)	0.81 (0.72–0.91)	0.64 (0.49–0.84)	—
**Radiotherapy**				$p = 0.03$
**Yes**	50.6 (35.9–64.3)	0.68 (0.63–0.73)	0.46 (0.40–0.53)	—
**No**	30.8 (27.8–37.8)	0.58 (0.53–0.64)	0.40 (0.34–0.46)	—
**Overall survival from time of 2L treatment**				
**Overall**	12.6 (10.0–14.6)	0.30 (0.24–0.38)	0.18 (0.13–0.26)	—
**Patient**				$p = 0.06$
**Recurrent**	15.9 (12.8–32.8)	0.38 (0.28–0.52)	0.21 (0.12–0.37)	—
**Advanced**	10.3 (8.0–13.2)	0.26 (0.19–0.35)	0.18 (0.12–0.27)	—
**Treatment type**				$p = 0.02$
**Platinum combination**	13.6 (11.6–21.4)	0.35 (0.26–0.48)	0.19 (0.11–0.32)	—
**Platinum monotherapy**	10.6 (7.6–NA)	0.30 (0.14–0.62)	0.30 (0.14–0.62)	—
**Non-platinum combination**	17.1 (12.7–NA)	0.36 (0.19–0.68)	0.36 (0.19–0.68)	—
**Non-platinum monotherapy**	8.0 (5.9–13.2)	0.23 (0.15–0.35)	0.12 (0.05–0.25)	—
**Overall survival from time of 2L treatment among patients on platinum regimen in 1L**				
**Overall**	10.4 (8.9–13.3)	0.28 (0.22–0.35)	0.17 (0.12–0.26)	—
**Patient**				$p = 0.5$
**Recurrent**	13.4 (9.3–37.4)	0.34 (0.22–0.53)	0.19 (0.09–0.40)	—
**Advanced**	10.3 (8.0–13.2)	0.26 (0.19–0.35)	0.18 (0.11–0.27)	—
**Rechallenge**				$p < 0.001$
**Rechallenged**	13.3 (11.2–20.9)	0.35 (0.27–0.46)	0.21(0.13–0.33)	—
**Not rechallenged**	6.4 (4.6–10.4)	0.16 (0.09–0.28)	0.13 (0.07–0.24)	—
**Treatment type**				$p = 0.002$
**Chemotherapy**	10.4 (8.9–13.3)	0.28 (0.22–0.35)	0.17 (0.12–0.26)	—
**Hormone**	NA (17.7–NA)	0.54 (0.38–0.77)	0.54 (0.38–0.77)	—
**Chemotherapy type**				$p = 0.02$
**Carboplatin**	10.4 (6.5–NA)	0.22 (0.08–0.59)	—	—
**Carboplatin + doxorubicin (liposomal)**	10.0 (8.9–NA)	—	—	—
**Carboplatin + paclitaxel**	13.3 (9.1–41.2)	0.39 (0.25–0.60)	0.13 (0.04–0.42)	—
**Doxorubicin (liposomal)**	6.7 (4.5–11.4)	0.10 (0.04–0.27)	—	—
**Overall survival from time of 3L treatment**				
**Overall**	11.0 (8.2–13.5)	0.28 (0.19–0.41)	0.11 (0.05–0.25)	—
**Patient**				$p = 0.6$
**Recurrent**	12.0 (8.0–27.6)	0.35 (0.22–0.57)	0.11 (0.03–0.38)	—
**Advanced**	11.0 (8.1–15.8)	0.21 (0.11–0.40)	0.11 (0.04–0.30)	—
**Treatment type**				$p = 0.01$
**Platinum combination**	20.1 (12.6–NA)	0.45 (0.27–0.75)	0.17 (0.05–0.54)	—
**Non-platinum monotherapy**	8.2 (5.3–12.3)	0.19 (0.10–0.36)	0.10 (0.04–0.27)	—

**Other**	<10	—	—	—

[1]*p*-value corresponding to chi-square test. 1L, first-line; 2L, second-line; 3L, third-line; CI, confidence interval; NA, not achieved.

Table S4. TTNT by line of therapy, patient type, and treatment type.

Strata	Median Survival (95% CI)	2-Year Survival (95% CI)	5-Year Survival (95% CI)	Log Rank Test p-value[1]
**TTNT from time of 1L treatment**				
**Overall**	19.9 (17.5-23.5)	0.45 (0.42–0.49)	0.30 (0.26–0.34)	—
**Patient**				$p = 0.3$
**Recurrent**	18.4 (14.1–24.2)	0.43 (0.37–0.50)	0.27 (0.20–0.35)	—
**Advanced**	21.3 (17.5–27.7)	0.47 (0.42–0.52)	0.32 (0.27–0.37)	—
**Treatment type**				$p < 0.001$
**Platinum combination**	19.6 (17.2–23.5)	0.45 (0.40–0.49)	0.30 (0.26–0.35)	—
**Platinum monotherapy**	7.1 (5.1–9.7)	0.15 (0.07–0.29)	0.02 (0.004–0.16)	—
**Non-platinum monotherapy**	8.3 (6.5–18.5)	0.25 (0.13–0.48)	0.08 (0.02–0.31)	—
**Non-platinum combination**	12.8 (9.3–NA)	0.44 (0.31–0.62)	0.30 (0.18–0.50)	—
**Hormone therapy**	62.5 (59.2–NA)	0.81 (0.72–0.91)	0.64 (0.49–0.84)	—
**TTNT from time of 2L treatment**				
**Overall**	7.0 (6.1–8.2)	0.22 (0.17–0.28)	0.19 (0.15–0.26)	—
**Patient**				$p = 0.4$
**Recurrent**	6.4 (5.6–7.8)	0.19 (0.12–0.30)	0.17 (0.10–0.29)	—
**Advanced**	7.6 (6.1–9.9)	0.23 (0.17–0.32)	0.20 (0.15–0.29)	—
**Treatment type**				$p < 0.001$
**Platinum combination**	9.6 (6.5–12.2)	0.22 (0.15–0.33)	0.19 (0.12–0.30)	—
**Platinum monotherapy**	6.4 (4.2–NA)	0.25 (0.11–0.570)	0.25 (0.11–0.57)	—
**Non-platinum combination**	4.6 (3.1–NA)	0.24 (0.10–0.57)	0.24 (0.10–0.57)	—
**Non-platinum monotherapy**	3.7 (3.2–5.5)	0.04 (0.01–0.14)	NA	—
**Hormone therapy**	NA (20.7–NA)	0.59 (0.43–0.80)	0.59 (0.43–0.80)	—
**TTNT from time of 2L treatment among patients on platinum regimen in 1L**				
**Overall**	7.1 (6.2–9.4)	0.22 (0.17–0.29)	0.19 (0.14–0.26)	—
**Patient**				$p = 0.5$
**Recurrent**	6.5 (5.2–10.0)	0.17 (0.09–0.33)	0.15 (0.07–0.30)	—
**Advanced**	7.6 (6.1–9.9)	0.24 (0.17–0.32)	0.21 (0.15–0.29)	—
**Treatment type**				$p < 0.001$
**Chemotherapy**	6.4 (5.3–7.7)	0.17 (0.12–0.24)	0.13 (0.09–0.20)	—
**Hormone**	NA (17.7–NA)	0.54 (0.38–0.77)	0.54 (0.38–0.77)	—
**Chemotherapy type**				$p = 0.003$
**Carboplatin**	6.4 (3.7–NA)	0.23 (0.09–0.61)	--	—
**Carboplatin + doxorubicin (liposomal)**	9.9 (7.1–NA)	--	--	—
**Carboplatin + paclitaxel**	9.4 (6.1–13.3)	0.20 (0.10–0.40)	0.13 (0.05–0.32)	—
**Doxorubicin (liposomal)**	3.9 (3.2–7.4)	0.05 (0.01–0.20)	--	—
**TTNT from time of 3L treatment**				
**Overall**	7.2 (6.0–10.2)	0.17 (0.11–0.28)	0.10 (0.05–0.20)	—
**Patient**				$p = 0.06$
**Recurrent**	9.0 (6.4–12.2)	0.22 (0.12–0.40)	0.13 (0.05–0.33)	—
**Advanced**	5.3 (4.7–10.5)	0.14 (0.06–0.30)	0.07 (0.02–0.24)	—
**Treatment type**				$p = 0.03$
**Platinum combination**	8.3 (5.1–26.6)	0.23 (0.10–0.54)	0.11 (0.03–0.42)	—
**Non-platinum monotherapy**	5.7 (4.7–9.9)	0.06 (0.01–0.21)	0.03 (0.004–0.19)	—
**Hormone monotherapy**	10.4 (6.0–NA)	0.44 (0.27–0.75)	0.30 (0.11–0.77)	—
**Other**	<10	--	--	—

[1]*p*-value corresponding to chi-square test.
